# *Malassezia* spp. Yeasts of Emerging Concern in Fungemia

**DOI:** 10.3389/fcimb.2020.00370

**Published:** 2020-07-28

**Authors:** Wafa Rhimi, Bart Theelen, Teun Boekhout, Domenico Otranto, Claudia Cafarchia

**Affiliations:** ^1^Dipartimento di Medicina Veterinaria, Università degli Studi “Aldo Moro”, Bari, Italy; ^2^Yeast Research, Westerdijk Fungal Biodiversity Institute, Utrecht, Netherlands; ^3^The Institute for Biodiversity and Ecosystem Dynamics (IBED), University of Amsterdam, Amsterdam, Netherlands; ^4^Shanghai Key Laboratory of Molecular Medical Mycology, Changzheng Hospital, Second Military Medical University, Shanghai, China; ^5^Faculty of Veterinary Sciences, Bu-Ali Sina University, Hamedan, Iran

**Keywords:** *Malassezia* spp., epidemiology, pathogenesis, fungemia, diagnosis, therapy, antifungal profile

## Abstract

*Malassezia* spp. are lipid-dependent yeasts, inhabiting the skin and mucosa of humans and animals. They are involved in a variety of skin disorders in humans and animals and may cause bloodstream infections in severely immunocompromised patients. Despite a tremendous increase in scientific knowledge of these yeasts during the last two decades, the epidemiology of *Malassezia* spp. related to fungemia remains largely underestimated most likely due to the difficulty in the isolation of these yeasts species due to their lipid-dependence. This review summarizes and discusses the most recent literature on *Malassezia* spp. infection and fungemia, its occurrence, pathogenicity mechanisms, diagnostic methods, *in vitro* susceptibility testing and therapeutic approaches.

## Introduction

*Malassezia* are lipid-dependent yeasts inhabiting the skin of healthy humans and other warm blooded animals. However, these yeasts may also act as opportunistic pathogens, causing dermatitis and otitis in animals, and dermatitis with (i.e., atopic dermatitis, folliculitis, and psoriasis) and without inflammation (*Pityriasis versicolor*) in humans (Guillot and Bond, 2020; Saunte et al., 2020). Besides their involvement in skin diseases, *Malassezia* spp. have increasingly been reported to cause severe systemic infections, especially among premature neonates and immunocompromised patients receiving parenteral nutrition (Miceli et al., 2011; Iatta et al., 2014a, 2018; Ilahi et al., 2017; Pedrosa et al., 2018). While fungemia caused by *Candida* species has been recognized as a cause of morbidity and mortality in hospitalized patients worldwide (Mellinghoff et al., 2018), the epidemiology of *Malassezia-*related fungemia remains largely underestimated most likely due to the difficulties in the isolation of these yeasts species due to their lipid-dependent growth (Iatta et al., 2018). Currently, the genus comprises 18 lipid-dependent species with a variable distribution on different hosts and pathologies (reviewed in Lorch et al., 2018; Guillot and Bond, 2020). Additionally, by using both fingerprinting methods and multigene sequence analysis, different *Malassezia* genotypes were identified as strictly related to the host, geographical origin, and/or clinical manifestations (Cafarchia et al., 2008, 2011b; Theelen et al., 2018). Several hypotheses have been proposed to explain the pathogenic mechanisms of these fungi, but the role of single species and genotypes in clinical manifestations remains to be elucidated (Theelen et al., 2018). In addition, scientific data suggest that *Malassezia* antifungal susceptibility profiles against azoles, amphotericin B (AmB) and terbinafine (TER) largely vary between *Malassezia* species or genotypes, thus influencing clinical management of patients (Theelen et al., 2018). This review summarizes and discusses the most recent literature on *Malassezia* fungemia, its occurrence, pathogenic mechanisms of the involved species, diagnostic methods, *in vitro* susceptibility testing and therapeutic approaches.

## The *Malassezia* Genus and Etiology of *Malassezia* Fungemia

Since the designation of the genus *Malassezia* by Baillon in 1889, the taxonomy has been updated and currently the genus comprises 18 lipid-dependent species with different genotypes showing variable pathology and distribution on different hosts (reviewed in Lorch et al., 2018; Guillot and Bond, 2020). All *Malassezia* species are lipid-dependent and, with the exception of *Malassezia pachydermatis*, do not grow on Sabouraud Dextrose Agar (SDA), which is most commonly used for culturing fungi in clinical labs. The genus *Malassezia* occurs on the skin of humans and animals but some species have only been observed on either humans or animals (reviewed in Guillot and Bond, 2020). Interestingly, *Malassezia* yeasts are the major component of the healthy human skin mycobiome (Findley et al., 2013; Oh et al., 2014; Wu et al., 2015). A recent phylogenetic study evaluating six genes suggested that the genus is deeply rooted in the Ustilaginomycotina and has a sister relationship with the Ustilaginomycetes and Exobasidiomycetes, assigning the genus its own class, Malasseziomycetes (Wang et al., 2014). Based on a phylogenomics study using 164 core eukaryotic genes, three main clusters were identified: Cluster A consisting of *M. furfur, M. japonica, M. obtusa, and M. yamatoensis*; Sub-cluster B1, with the most abundant human skin inhabitants *M*. *globosa* and *M. restricta*; Sub-cluster B2 consisting *of M. sympodialis, M. dermatis, M. caprae, M. equina, M. nana*, and *M. pachydermatis*; and Cluster C forming a basal lineage with *M. cuniculi and M. slooffiae* (Wu et al., 2015). Four more species have been described since then: *M. brasiliensis* and *M. psittaci* from parrot (Cabañes et al., 2016), *M. arunalokei* from human scalp (Honnavar et al., 2017), and *M*. *vespertilionis* from bat (Lorch et al., 2018). Multiple genotypes of a species can colonize the same patient (Cafarchia et al., 2008; Machado et al., 2010; Ilahi et al., 2017) but some genetic types might be linked to a particular body site or pathology thus indicating an affiliation of *Malassezia* genotypes with host, geographical origin and/or clinical manifestations (Cafarchia et al., 2008, 2011a,b; Ilahi et al., 2017). In particular, amplified fragment length polymorphism (AFLP) patterns of *M*. *furfur* skin isolates from Ontario, Canada clustered separately from mainly European references from other body sites, suggesting geographical or ecological/clinical variability in the species (Gupta et al., 2004). Sequence analysis of the intergenic spacer (IGS1) distinguished specific *M. globosa, M. restricta*, and *M. pachydermatis* variants in seborrheic dermatitis, atopic eczema, and on healthy skin of humans and animals (Sugita et al., 2003, 2004; Kobayashi et al., 2011). Moreover, sequence analyses of the LSU rDNA showed distinct *Malassezia* spp. subtypes on different host species (Gaitanis et al., 2012). Multilocus sequence analysis that included the D1/D2 domains of LSU rDNA, the chs2 gene, and the ITS1 region grouped *M. pachydermatis* strains from skin of healthy dogs and from skin lesions in three main genotypes (A, B, and C) with eight ITS1 subtypes. Genotype B included isolates from dogs of European origin and appeared to be present on healthy dog skin, without producing phospholipase. The A and C genotypes and their subtypes seemed to be predominantly associated with skin lesions and their isolates showed high phospholipase production (Cafarchia et al., 2008; Machado et al., 2010). Similarly, IGS1 subtypes 3C and 3D displayed high phospholipase production and were more frequently isolated from skin lesions of dogs with atopic dermatitis (Kobayashi et al., 2011). Only three *Malassezia* species have been described to cause bloodstream infections: *M*. *furfur, M*. *pachydermatis*, and *M*. *sympodialis*. Interestingly, AFLP analysis or ITS sequences showed that only one main *M. furfur* or *M. pachydermatis* genotype seems to be involved in blood stream infections in immunocompromised hosts (Theelen et al., 2001; Kaneko et al., 2012; Ilahi et al., 2017). All these *Malassezia* genotypes might also colonize the skin of patients or of hospital staff which might represent the driver for these systemic infections (Theelen et al., 2001; Gupta et al., 2004; Kaneko et al., 2012). With respect to the assessment of antifungal microbiological profiles, a reference method has not yet been developed for these yeasts and the culture media, inoculum sizes, incubation times, and the criteria used to determine MIC endpoints differ among studies. However, evidence suggested that *Malassezia* antifungal susceptibility profiles against azoles, AmB and TER vary according to the *Malassezia* species, regardless of culture medium or other conditions employed. *M. sympodialis* and *M. pachydermatis* are the most susceptible and *M. furfur* and *M. globosa* the least susceptible species to azoles, AmB and TER (Rojas et al., 2014; Cafarchia et al., 2015; Pedrosa et al., 2019b). Itraconazole (ITZ) and ketoconazole (KTZ) were the most active drugs for all *Malassezia* species, and fluconazole (FLZ), voriconazole (VOR) and AmB the least active (Rojas et al., 2014; Cafarchia et al., 2015; Pedrosa et al., 2019b). Interestingly, antifungal profiles may also vary in relation to genotype (Sugita et al., 2005; Cafarchia et al., 2012). In particular, isolates derived from animal skin lesions and belonging to a unique genotype, showed reduced susceptibility to azoles when compared to genotypes associated with healthy skin (Cafarchia et al., 2008).

## Epidemiology of *Malassezia* Yeasts and Fungemia

The epidemiology of *Malassezia* fungemia has not been well-investigated until now due to the scant surveillance studies with this focus (see Table 1). *Malassezia furfur, M. sympodialis*, and *M. pachydermatis* are the only *Malassezia* species isolated from bloodstream infections to date (Table 1). Some authors propose that the fungal density on the skin as well the host immunological competence might be driving factors influencing their pathogenic role (Wheeler et al., 2017; partially reviewed in Theelen et al., 2018). *Malassezia* bloodstream infections have been described in adult and child immunocompromised patients, and neonates (Table 1). According to general knowledge, the main ecological niche of *Malassezia* yeasts is human and animal skin, and they represent 50 to 80% of the total human skin mycobiome (Nagata et al., 2012; Findley et al., 2013; Gupta et al., 2014). Therefore, it would make sense to consider the skin mycobiome as a potential reservoir and point d'entrée for bloodstream infections. More is currently known about the adult human skin mycobiome but little information is available about the child and neonatal skin mycobiome. Even if *Malassezia* skin colonization may be the result of both maternal and environmental sources, data suggests that colonization of human skin begins immediately after birth and *Malassezia* species distribution varies with age (Jo et al., 2016; Ward et al., 2018). The limited data currently available about the cutaneous mycobiomes in preterm and term neonates shows that *Malassezia* species distribution on skin of neonates and children varies between studies, but *M. globosa, M*. *furfur, M*. *sympodialis*, and *M*. *restricta* seem to be the most prevalent species described (Bernier et al., 2002; Zomorodain et al., 2008; Jang et al., 2009; Gupta et al., 2014; Prohic et al., 2014; Jo et al., 2016; Paul et al., 2019). Variation between studies could be the result of methodological differences such as number of subjects studied, DNA extraction, target region selection and PCR-conditions, and data processing and interpretation; but also geographical and host-specific differences may explain study discrepancies. The study of Paul et al. determined *M*. *restricta* to be the most abundant *Malassezia* species in both term and preterm neonates (*n* = 30), similar to adult skin (Paul et al., 2019). Interestingly, this species has thus far not been linked to bloodstream infections. *Malassezia* colonization increases quickly after birth but a very important shift, making *Malassezia* the most predominant genus of the human skin mycobiome, takes place during adolescence and has been linked to lipid composition shifts of the skin (Grice and Segre, 2011; Findley et al., 2013; Jo et al., 2016). In adults, *M*. *restricta* and *M*. *globosa* seem to be the dominating *Malassezia* species on the skin, followed by *M*. *sympodialis*, depending on body site (Findley et al., 2013; Wu et al., 2015). Geographical factors may influence *Malassezia* species distribution on healthy human skin (Leong et al., 2019; Saunte et al., 2020). In addition, the proportion of *Malassezia* species isolated from the skin varies considerably among different medical conditions (Saunte et al., 2020). As the two most abundant *Malassezia* species on healthy human adult skin have not been linked to *Malassezia* bloodstream infections, studies focusing on genetic and functional differences between cells of *Malassezia* species involved in bloodstream infections and ones that are not, in addition to potentially selective host parameters, could help explain this. Recent molecular studies highlighted the genetic variability among single species implicated in both skin and systemic infections (Theelen et al., 2001; Kaneko et al., 2012; Gupta et al., 2014), suggesting pathogenicity variation at species and sub-species levels. *Malassezia pachydermatis* is mainly known from animals and is not commonly associated with human skin colonization yet in multiple cases it was linked to *Malassezia* bloodstream infections (Table 1).

**Table 1:** *Malassezia* yeasts fungemia: yeast species, risk factors, diagnosis, treatment, and outcome.

**References**	**Yeast species**	**Hosts and number**	**Cause of admission**	**Risk factors**	**Diagnosis**	**Treatment protocol**	**Length of treatment**	**Outcome**
Redline and Dahms (1981)	*M. furfur*	Preterm neonate/1	Respiratory distress syndrome	CVC, Preterm neonates, TPN	Histology/cytology	AmB + Flucytosine	NR	Dead
Hassall et al. (1983)	*M. furfur*	Children/1	Fever, vomiting	CVC, bowel syndrome, urokinase therapy	Counter current immune-electrophoresis	CVC removal, AmB	42 days	Alive
Powell et al. (1984)	*M. furfur*	Preterm neonate/4 Children/1	Fever, SEPSIS interstitial pneumonia, elevated neutrophil band counts, and thrombocytopenia	CVC, TPN, preterm neonates, prolonged hospitalization, pneumonia	CCVC tip culture on lipid media	CVC removal	NR	Alive
Redline et al. (1985)	*M. furfur*	Preterm neonate/3 Children/2 Adult/2	Fever, thrombocytopenia in neonates, respiratory distress	CVC, TPN, preterm neonates, severe gastrointestinal disease and immunosuppression	Histology/cytology/blood and CVC tip cultures on lipid media	Discontinuation of the lipid administration, AmB (0.5–1 mg/kg/d), or AmB (40 mg/day), Flucytosine every 6–8 h	30–35 days	Dead (preterm neonates) Alive (children and adults)
Alpert et al. (1987)	*M. furfur*	Preterm neonate/6 Children/1	Fever, respiratory distress asymptomatic (2 infants)	CVC, TPN, multiple potential causes for immunosuppression	Blood culture on lipid media	CVC, AmB alone or with fluorocytocine	5–42 days	2 Dead due to unrelated causes and 5 alive
Aschner et al. (1987)	*M. furfur*	Children/2	Fever, pulmonary diseases, thrombocytopenia	Apnea, bradycardia, pulmonary deterioration, thrombocytopenia	CVC tip on lipid media	CVC removal	NR	1 Dead 1 Alive
Dankner et al. (1987)	*M. furfur*	Preterm neonate/5 Adult/2	Fever, thrombocytopenia, hyperbilirubinemia	CVC, Premature neonates, prolonged hospitalization, tpn, broad-spectrum antibiotics, multiple potential causes for immunosuppression	Blood culture bottles on lipid media	AmB alone or AmB and flucytosine	4 days−6 weeks	Alive
Garcia et al. (1987)	*M. furfur*	Adult/2	Fever and leukocytosis	CVC, TPN, Broad-spectrum antibiotics, multiple potential causes for immunosuppression	Blood culture on lipid media	Discontinuation of the lipid administration AmB (total dose 665 mg)	28 days	Dead
Wurtz and Knospe (1988)	*M.furfur*	Adult /1	Fever	Multiple potential causes for immunosuppression	Blood culture on lipid media	AmB followed by cephalexin and KTZ	18–25 days	Alive
Shek et al. (1989)	*M. furfur*	Preterm neonates/3	NR	NR	Histology	NR	NR	Dead due to *Malassezia* septicemia
Surmont et al. (1989)	*M. furfur*	Preterm neonates/6	Fever, pulmonary diseases, leucocytosis, thrombocytopenia	CVC, total parenteral nutrition, preterm neonates, broad-spectrum antibiotics, multiple potential causes for immunosuppression	CVC culture on lipid media	CVC removal, discontinuation of the lipid administration miconazole (13–30 mg/kg per d) alone or in combination with or cloxacillin and netilmicin	10–15 days	1 Dead 5 Alive
Masure et al. (1991)	*M. furfur*	Preterm neonates/1	Leukemia	CVC	Blood culture on lipid media	NR	NR	NR
Weiss et al. (1991)	*M. furfur*	Preterm neonates/1	Preterm neonates	CVC, total parenteral nutrition, preterm neonates	Blood culture on lipid media	Discontinuation of the lipid emulsion without CVC Removal	–	Alive
Welbel et al. (1994)	*M. pachydermatis*	Preterm neonates /5	Thrombocytopenia	CVC, TPN, interavenous lipid, preterm neonates (interveuticular hemmorhage, sepsis, respiratory distress syndrome), prolonged hospitalization	Blood culture	NR	NR	NR
Barber et al. (1993)	*M. furfur*	Adults /4 children/3	Fever, leukocytosis, cough, apnea, myalgia, nausea/vomiting	CVC, Multiple potential causes for immunocompromise	CVC culture on lipid media	AmB (1 mg/kg/d)	NR	2 Dead due to unrelated causes 5 Alive
Shparago et al. (1995)	*M. furfur*	Adult/1	Fever, chills, dyspnea, pleuritic chest pain, and multiple bilateral pulmonary nodular infiltrates	CVC, TPN	Lysis-centrifugation fungal blood cultures on lipid media	CVC removal discontinuation of the lipid emulsion, and AmB (300-mg total cumulative dose)	>2 weeks	Alive
Schoepfer et al. (1995)	*M. furfur*	children/3	T cell lymphoma. meningeal relapse bone marrow transplantation.	CVC, TPN with lipids, broad-spectrum antibiotic and corticosteroid treatment,	Cytology	AmB, flucytosine FLZ	NR	Alive
Chang et al. (1998)	*M. pachydermatis*	Preterm neonates/8	Fever intubation or reintubation and tachycardia	CVC, TPN with Lipids,	Blood cultures	AmB for at least 10 days	NR	1 dead 7 alive
Morrison and Weisdorf (2000)	*M. furfur*	Children/1 Adult/1	Fever, Hurler's allogeneica trasplant	CVC, TPN with Lipids,	Histology, dupont isolator system on lipid media	CVC removal discontinuation of the lipid emulsion	NR	1 dead due to unrelated causes 1 alive
Schleman et al. (2000)	*M. pachydermatis*	Adult /1	Fever	CVC, TPN with lipids, multiple abdominal surgeries	Blood culture bottles	CVC Removal AmB	NR	Alive
Chryssanthou et al. (2001)	*M. pachydermatis*	Preterm neonates/8	Fever, respiratory distress syndrome	Preterm neonates, TPN	BACTEC blood culture bottles	L-AmB (1 mg/Kg/d), Flucytosine (8 mg/Kg/d PO or EV), FLZ (50–150 mg kg/d)	21–35 days	Alive
Kikuchi et al. (2001)	*M. sympodialis*	Adult/1	Gastric cancer	Fever elevated leukocyte counts and C-reactive protein	NR	NR	NR	NR
Chu and Lai (2002)	*M. furfur*	Adult/1	Fever	Broad-spectrum antibiotic corticosteroid treatment,	Blood culture bottles	AmB (0.7 mg/kg/d for 10 days), FLZ (200 mg daily for 14 days)	24 days	Alive
Rosales et al. (2004)	*M. furfur*	Preterm neonate/1	Hypotension, Thrombocytopenia, High level C-reactive protein apnea, temperature instability, Bilious residual	CVC, Parenteral nutrition, preterm neonate (low birth-weight), chronic lung disease, necrotizing, enterocolitis intraventricular	Histology, radiographically/ photomicrograp	AmB	26 days and 2 days	Died
Giusiano et al. (2006)	*M. furfur M. sympodialis*	Adult/2	Oncology	NR	CVC tips cultures	Nr	NR	NR
Oliveri et al. (2011)	*M. furfur*	Preterm neonate/1	Fever, gastrointestinal disturbs, cyanosis	CVC, Parenteral nutrition, prolonged hospitalization, antibiotic treatment, ileostomy surgery	Lysis-centrifugation blood culture on Dixon	CVC removal L-AmB (4 mg/Kg/d)	45 days	Alive
Iatta et al. (2014a)	*M. furfur*	Preterm neonates/6 (3 with FLZ prophylaxis) and children/2	Fever, apnea, high level c reactive protein, abdominal distension, thrombocytopenia	CVC, Parenteral nutrition, Preterm neonates, Prolonged hospitalization, Neonatal asphyxia, Abdominal surgery (infants)	Lysis-centrifugation blood culture on Dixon	CVC removal L-AmB (2.5–5 mg/Kg)	6–20 days	Alive
Al-Sweih et al. (2014)	*M. pachydermatis*	Preterm neonate/1	Fever, respiratory distress syndrome	CVC, Preterm neonates TPN, necrotizing enterocolitis	BACTEC blood culture bottles	L-AmB	7 days	Alive
Choudhury and Marte (2014)	*M. pachydermatis*	Adult with posaconazole prophylaxis/1	Fever	CVC, acute myeloid leukemia, chemotherapy	BACTEC blood culture bottles	CVC removal L-AmB (1 mg/Kg/d)	NR	Alive
Aguirre et al. (2015)	*M. sympodialis*	Children /1	Fever, respiratory distress with hypoxemia	CVC, History of prolonged hospitalization, broad-spectrum antibiotic treatment Abdominal surgeries, viral infections	Lysis-centrifugation blood culture on Dixon	CVC removal, L-AmB (1 mg/Kg/d)	21 days	Alive
Roman et al. (2016)	*M. pachydermatis* + mycobacteria	Adult/1	Fever, pneumonia, dyspnea	CVC, Bacteremia, Leprosis	BACTEC blood culture bottles	CVC removal, AmB (5 mg/Kg/d) and nafcillin (400 mg IV d)	7 days	Alive
Iatta et al. (2018)	*M. furfur*	Preterm neonates/9	Fever, respiratory distress, elevated or depressed leukocyte count, increased C-reactive protein levels, thrombocytopenia	CVC, Preterm neonates TPN	CVC tips cultures on lipid media	NR	NR	1 Died and 8 Alive
Pedrosa et al. (2018)	*M. furfur*	Preterm neonates/1 and Adult /2 with fluconazole prophylaxis	Fever	CVC, Broad-spectrum antibiotic and glucocorticoid therapies, Auto-immune diseases, Carcinoma	BACTEC blood culture bottles and (MALDI-TOF-MS)	CVC removal, FLZ (10 mg/kg/d) and/or L-AmB (3 mg/kg/d) or (5 mg/kg/d) for Adults	50 days	2 Dead (adults) 1 Alive
Chen et al. (2019)	*M. furfur*	Preterm neonates with fluconazole prophylaxis/1	Fever, apnea, bradycardia, thrombocytopenia	CVC, TPN with Lipids Premature infant (Low birth-weight).	Blood culture in media with lipid	CVC removal, AmB (1 mg/kg/d)	NR	Alive
Lee et al. (2019)	*M. pachydermatis*	Adult/1	Intraabdominal abscess	CVC, TPN with Lipids, Multiple potential causes for immunocompromised, Gastric cancer, Chemotherapy	Blood culture on 5% on Sabouraud dextrose agar.	AmB	2 days	Dead due to unrelated causes
Huang et al. (2020)	*M. pachydermatis*	Preterm neonates with fluconazole prophylaxis/4	Respiratory distress syndrome, low Apgar scores	CVC, broad-spectrum antibiotics, TPN with Lipids, low birth weight	BACTEC blood culture bottles	CVC removal (2/4), AmB	14–21 days	Alive
Chow et al. (2020)	*M. pachydermatis*	Preterm neonates with fluconazole/3	CLABSI, necrotizing enterocolitis, peritonitis, infected urinoma	CVC, Broad-spectrum antibiotics, TPN with Lipids, Preterm neonate (low birth-weight)	CVC culture	NR	NR	Alive

*NR, not reported; CVC, central venous catheter; TPN, total parenteral nutrition; AmB, Amphotericin b; L-AmB, Liposomal Amphotericin b; FLZ, fluconazole; CLABSI, central line-associated bloodstream infection*.

A study evaluating cats and dogs (107 healthy and 123 with chronic otitis externa), showed that occurrence and population size of *M*. *pachydermatis* increased according to the presence in skin lesions, not only in affected areas of the skin but also at other sites without detectable skin lesions, suggesting that the yeasts could be easily transmitted from site to site because of scratching induced by pruritus (Cafarchia et al., 2005). This finding is also relevant from a zoonotic point of view since the yeasts may be mechanically transmitted from dogs to their owners (Morris et al., 2005) and subsequently can cause health problems as previously reported from an intensive care nursery (Chang et al., 1998). A similar transmission route—animal to human or human to human—from health care worker or family member for *M*. *furfur* or *M*. *sympodialis* may also occur and should be further explored in future studies. Even when these species are part of the stable mycobiomes of *Malassezia* bloodstream infection patients, isolates causing the infection could belong to different genotypes, transmitted from external sources. The first case of *Malassezia* spp. as a pathogen in bloodstream infection and sepsis was reported in 1981 by Redline and colleagues (Table 1). Until now, a total of 118 cases were published, but only three surveillance studies (Table 1). *Malassezia furfur* was the most encountered species with 82 cases, followed by *M*. *pachydermatis* (33 cases), and only three cases of *M*. *sympodialis* fungemia have been described. To date, *Malassezia* fungemia cases were observed with highest incidence in neonates (82 cases), followed by 23 in adults and 17 in children. Numerous cases were reported in the past two decades, particularly in neonates and infants receiving intravenous lipids. In our 1-year survey on yeast fungemia involving 290 neonatal and 17 pediatric patients with intravascular catheters, lipid parenteral nutrition, prolonged ward stay, and surgery, were evaluated. This diagnostic survey on bloodstream infections (BSIs) resulted in a higher prevalence of *M. furfur* (2.1%) than *Candida* spp. (1.4%) and suggested that *Malassezia* BSIs might be underestimated, due to improper diagnosis (Iatta et al., 2014a). The surveillance study was repeated 4 years later, and a total of 202 neonatal patients with intravascular catheters were enrolled (Iatta et al., 2017). A total of 10 cases of BSIs were registered, thus suggesting the relevance of these yeasts in catheter mediated fungemia (Iatta et al., 2018). Incubator, sheets and the skin of patient or hospital staff may represent potential sources of *Malassezia* infection. Recently, lipid infusion type, phototherapy light sources, central venous catheter placement, and prophylactic fluconazole were proposed as risk factors affecting *Malassezia* colonization and /or fungemia more than candidemia (Chen et al., 2019).

## Pathogenesis

Almost all-available research to date focuses on *Malassezia* virulence factors linked to skin and pathogenesis for *Malassezia* BSIs has hardly been studied, likely due to the relatively low number of cases when compared to skin diseases. With an increasing number of cases in recent years and the likely underestimation of *Malassezia* BSIs as a result of the use of standard culture media without lipid supplementation in the clinic, future studies will hopefully address some of the missing knowledge. Here, we focus on known virulence factors of the three fungemia causing *Malassezia* species (i.e., *M. pachydermatis, M. furfur*, and *M. sympodialis*) and discuss findings, even if they were derived from skin. Virulence factors, to some extent, may be of a more general nature and could potentially also be relevant for bloodstream infections. Several hypotheses have been proposed to explain the pathogenic behavior of these fungi. The relationship between host and *Malassezia* metabolism seems to be key for the understanding the pathogenesis of infection (Cafarchia and Otranto, 2004; Velegraki et al., 2015; Theelen et al., 2018).

Various *Malassezia* cell characteristics may be involved in BSI pathogenesis. *Malassezia* spp. have cell walls with a very thick multi-layered structure that may protect them from different environmental stresses and have been described to help evade phagocytosis (Celis et al., 2017a). Biofilm formation has been linked to increased drug resistance and virulence. *M. furfur, M. sympodialis*, and *M. pachydermatis* are able to form biofilms, a process that seems to be strain dependent (Figueredo et al., 2013; Angiolella et al., 2017; Pedrosa et al., 2019a). In particular, *M. pachydermatis* strains isolated from dogs with and without skin lesions were able to form biofilms with variable extracellular matrix (ECM) quantity and structure depending on the sources (Figueredo et al., 2013). Accordingly, a structural heterogeneity of biofilm was found between those formed by *M. furfur* and *M. sympodialis* isolates, with both species exhibiting yeast aggregates in multilayer clusters but with a denser entrapment by a more gelatinous ECM in case of *M. furfur* biofilms (Pedrosa et al., 2019a). Biofilm formation and the extracellular matrix generation were responsible for the emergence of antifungal resistance (Figueredo et al., 2013; Angiolella et al., 2017). The biofilm formation was well-correlated with hydrophobicity, adherence, and phospholipase production of pathogenic *M. pachydermatis* and *M. furfur* cells, which may help explaining the change from a commensal to a pathogenic phenotype of these organisms (Figueredo et al., 2013; Angiolella et al., 2017). *Malassezia furfur* colonization of central venous catheters was already observed in 1994 (Sizun et al., 1994) and the ability *M*. *pachydermatis* to form biofilms on catheter surfaces in the laboratory was confirmed in another study (Cannizzo et al., 2007). Though not observed frequently, *Malassezia* yeasts are able to produce mycelium, a feature first suggested from an observation of hyphae in the scales of PV. Later, researchers managed to also obtain a mycelial phase for *M*. *furfur in vitro* (Saadatzadeh et al., 2001). In a recent review, the importance of morphological switching for fungal pathogens was highlighted: shape-shifting between different morphologies allows fungi to adapt to different host environments. The authors suggest that to understand pathogenesis mechanisms, it is crucial to establish how fungal morphology impacts virulence strategies (Min et al., 2020). It would be useful to further investigate *Malassezia* morphology switching and its potential relevance in bloodstream infections.

It has been shown that μ-opioid receptors (MORs) are present on *M. pachydermatis* cell membranes, having a role in modulating the phospholipase activities (Cafarchia et al., 2010). In animals without lesion MORs were expressed as dimers with other opioids thus resulting in an inactive form (Cafarchia et al., 2010). The high concentration of beta-endorphin normally present on lesioned skin of hosts (Pan, 2005; Honnavar et al., 2017) influenced the expression of MOR in their active form, thus favoring phospholipase production (Cafarchia et al., 2010). Increased phospholipase activity may be linked to the appearance of skin lesions and in some cases septicaemia (Cafarchia and Otranto, 2004; Vlachos et al., 2013).

*Malassezia* yeasts also produce esterases, lipases, and proteases, of which the latter have a crucial role in interactions with the host and microbial community such as *Staphylococcus aureus* thus making it unfavorable for colonization (Chen and Hill, 2005; Li et al., 2018; Tee et al., 2019). Additionally, *M. furfur* secretes aspartyl proteases, capable of degrading a wide range of human skin associated extracellular matrix (ECM) protein, and might also be able to modify the skin environment potentially interfering with wound re-epithelization (Poh et al., 2020). These enzymes could on one hand contribute to pathogenesis but on the other hand have a protective function, leading to a potentially very complex role for *Malassezia* on and in the human host. Lipases particularly have been considered virulence factors of *Malassezia* yeasts since they may be involved in the invasion, colonization, persistence and proliferation within host tissues (Petrokilidou et al., 2019). The first *Malassezia* gene encoding an extracellular lipase was identified in *M. furfur* and was designated as LIP1. Subsequently, orthologs were identified in *M. pachydermatis, M. globosa*, and *M. restricta*. *Malassezia* species possess multiple genes encoding putative lipases (from 9 to 14 depending on the species) that are differently involved in various skin disorders (Park et al., 2017; Tee et al., 2019). Interestingly, the lipase and phospholipase activities of *Malassezia* yeasts vary according to the species and they are implicated in both skin diseases and fungemia. In particular, *M. furfur* strains causing fungemia showed very high lipolytic enzyme activity thus suggesting that parenteral lipid emulsions may play an important role in modulating the growth and pathogenicity of *Malassezia*-yeasts in sepsis (Kaneko et al., 2012). *Malassezia* strains from skin disorders produce metabolites, such as indirubin and indolo[3,2-b] carbazole (ICZ) which are associated with carcinogenesis, immune regulation and mediation of ultraviolet radiation (UVR) damage (Gaitanis et al., 2011; Theelen et al., 2018). Recently, it has been shown that both *M. furfur* and *M. sympodialis* are able to produce nanovesicles enriched with allergens and/or proteins that interact with keratinocytes and monocytes, thus causing and maintaining the inflammation (Johansson et al., 2018; Zhang et al., 2019). In particular, nanovesicles produced by *M. sympodialis* (MalaEx) are also able to activate human keratinocytes causing an enhanced intercellular adhesion molecule-1 (ICAM-1) expression, which can cause an attraction of immune competent cells, thus causing host cutaneous defense to *M. sympodialis* (Vallhov et al., 2020). So far, the function of these extracellular vesicles for *Malassezia* spp. has only been studied in relation to skin but various studies in other fields related to similar kinds of nanovesicles show that their function in cell-to-cell communication may be much broader than that. For example, a study on human pathogen *Cryptococcus gattii*, showed that vesicles were taken up by macrophages of the infected host, allowing long distance pathogen-to-pathogen communication resulting in virulence enhancement (Bielska et al., 2018). It would be interesting to explore whether extracellular vesicles might also play a role in *Malassezia* BSIs.

In order to gain better understanding of *Malassezia* pathogenesis in general, and in BSIs in particular, it is important to assess known *Malassezia* virulence factors, as well as investigate potentially unknown factors, and perform comparative studies between BSI-derived isolates and skin isolates. In recent years, many useful new tools have been developed for this purpose. Although to date, no model systems for studying systemic *Malassezia* infections have been described, recent advances for other *Malassezia*-affected areas of the human body may offer useful insights for future application to studying host-pathogen interactions in BSIs. Sparber et al., reported of a murine epicutaneous infection model that allowed studying the interaction of *Malassezia* with mammalian skin *in vivo* (Sparber and LeibundGut-Landmann, 2019; Sparber et al., 2019) and in the same year the association of *Malassezia* with Crohn's disease was reported using mouse models (Limon et al., 2019). Performing *Malassezia* host-pathogen interaction studies in model systems such as the mouse may sometimes be difficult, but with the recent establishment of an *in vivo* infection model using *Galleria mellonella*, host-pathogen interaction studies are becoming more accessible. The *G*. *mellonella* model has multiple advantages, such as the absence of ethical hurdles, low cost, ease of use, yet the immune response has similarities with the human system (Torres et al., 2020). In addition, recently developed *Malassezia* transformation systems can aid in studying the role of specific genes and virulence factors in *Malassezia* pathogenicity. *Agrobacterium tumefaciens* mediated transformation systems for the BSI-relevant *Malassezia* species were developed, allowing direct gene manipulation to better understand gene function (Ianiri et al., 2016; Celis et al., 2017b). With the recent improvements to the CRISPR/Cas9 strategy, this gene editing system has already contributed to various fungal virulence studies (Malavia et al., 2020), and also its first application for research in functional *Malassezia* genetics has recently been reported (Ianiri et al., 2019).

## Diagnosis

Since multiple *Malassezia* species and/or genotypes with varying antifungal susceptibility profiles may cause unique or similar pathologies, serious concern about the diagnostic procedures and antifungal treatment has been raised. Isolation and enumeration of *Malassezia* cells from clinical specimens remain a challenge because of their lipid dependency. Although microscopy of swab specimens is useful for diagnosing animal and human dermatitis, a more accurate etiological diagnosis is needed in high-risk patients (Iatta et al., 2018). Clinical features, laboratory markers, strategies of patient management, and outcomes of *Candida* and *Malassezia* fungemia do not differ. In some studies*, M. furfur* fungemia appeared earlier than candidaemia (average day 26 vs. day 42), most likely due to its exogenous origin. In addition, duration of *Malassezia* fungemia is longer than candidaemia, most likely due to the late removal of the central venous catheters (CVC) and also as a result of the lower efficacy of the antifungal therapy (Iatta et al., 2014a, 2018). The first signs causing suspicion of *Malassezia* fungemia usually are bloodstream infections manifested in fever of unknown origin in hospitalized and severely immunocompromised patients with CVC (i.e., preterm neonates or cancer patients) or in patients with pulmonary distress (Table 1) (Morrison and Weisdorf, 2000; Iatta et al., 2015, 2018). *Malassezia* fungemia should however be confirmed with the laboratory isolation of the responsible agent and identification by molecular means (Morrison and Weisdorf, 2000; Iatta et al., 2015, 2018; Pedrosa et al., 2018). In literature *Malassezia* yeasts were mainly isolated by culturing blood or CVC-tip directly on specific media and mainly by using the Isolator system (Table 1). It has been shown that the automated blood culture system BacT/Alert is not suitable for detecting *M. furfur* fungemia (Campigotto et al., 2016; Iatta et al., 2018). Out of 9 *M. furfur* fungemia cases reported by using BacT/Alert bottles only 1 case of *M. furfur* fungemia was detected whereas all the *M. pachydermatis* cases were easily detected using the above mentioned method, since this species grows on the media included in the bottles (Iatta et al., 2018, Table 1). Interestingly, it has been shown that human blood has a toxic effect on yeast growth and the addition of 3% of palmitic acid in the bottle might be able to overcome the inhibitory effect of both small (0.5 ml) and larger (3 ml) volumes of blood, thus favoring *Malassezia* growth (Nelson et al., 1995). Finally, although molecular tools have been used to detect *Malassezia* yeasts from biological samples no studies were performed to molecularly diagnose *Malassezia* directly from blood, thus culture remains the gold standard to isolate and identify the yeasts. This method is also suitable for yeast quantification, viability assessment, and genotyping and eventually to test the antifungal susceptibility profile of the isolated species (Peker et al., 2018). Generally, clinics use standard culture media without lipid supplementation, likely leading to underdiagnosis of both *M*. *furfur* and *M*. *sympodialis* in BSIs. It is recommended to carefully assess *Malassezia* BSI risk factors and apply the use of lipid-rich culture media when *Malassezia* spp. may be the causative agent.

## Therapy, Antifungal Profile, and Probable Resistance Phenomena

For treatment of *Malassezia-*related infections, azoles, and the polyene AmB are frequently employed, both in humans and animals. Topical antifungal agents (mainly azoles) are adequate for the management of localized skin lesions, while systemic ITZ or FLZ for severe skin diseases (Bond et al., 2020; Saunte et al., 2020). For catheter-related *Malassezia* infections, there are only recommendations, and patients were usually treated with catheter removal, discontinuation of lipid infusion and administration of antifungal drugs such as FLZ, AmB, and or VOR (Arendrup et al., 2014; Table 1). Amphotericin B is effective in the treatment of *Malassezia* systemic infections, both in preterm infants and adults, but both FLZ and posaconazole (POS) fail to prevent *Malassezia* fungemia (Table 1). Usually, about 24 days of AmB treatment might be useful for a positive outcome of *Malassezia* fungemia but the length of the treatment might be different depending on the *Malassezia* species (Table 1). *M. pachydermatis* fungemia resolve more quickly than *M. furfur* fungemia (about 14 vs. 24 days, Table 1).

Despite attempts to treat these fungal infections, a trend toward recurrence is often observed in humans and animals with dermatitis (Negre et al., 2009; Bond et al., 2010, 2020; Saunte et al., 2020). Moreover, the induction of *in vitro* FLZ resistance in *M. pachydermatis* as well as the clinical evidence of treatment failure with TER in patients with *pityriasis versicolor* and, with KTZ in dogs with otitis (Kim and Pandya, 1998; Gupta et al., 2014; Kim et al., 2018) or with FLZ or POS in preventing *M. furfur* fungemia in humans, suggested the occurrence of drug resistance phenomena in these yeast species (Choudhury and Marte, 2014; Iatta et al., 2014a; Pedrosa et al., 2018; Chen et al., 2019). Antifungal susceptibility test methods have not yet been standardized, neither by the Clinical and Laboratory Standards Institute (CLSI) nor by the European Committee on Antimicrobial Susceptibility Testing (EUCAST) (Arendrup et al., 2014), resulting in the absence of clinical breakpoints for these yeasts species. A recent study showed that drug efflux pumps (EPMs) are involved as defense mechanisms to azole drugs in *Malassezia* yeasts (Iatta et al., 2017). By using a broth microdilution chequerboard analysis, the *in vitro* efficacy of azoles in combination with EPMs (i.e., haloperidol-HAL, promethazine-PTZ, and cyclosporine) was evaluated. The MICs of FLZ and VOR of *Malassezia* spp. decreased in presence of sub-inhibitory concentrations of HAL and/or PTZ, and a synergistic effect was observed only in strains with FLZ MIC ≥ 128 μg/mL for *M. furfur*, FLZ MIC ≥ 64 μg/mL for *M. pachydermatis*, and VOR MIC ≥ 4 μg/mL in both *Malassezi*a spp., suggesting that the above FLZ and VOR MIC values might be considered the cut-off to discriminate susceptible and resistant strains (Iatta et al., 2017). Finally, the *in vitro* susceptibility of *Malassezia* for echinocandins suggests that this genus is intrinsically resistant to these drugs. Indeed, MIC > 32 μg/mL were usually recorded for *M. pachydermatis* and *M. furfur* regardless of the employed CLSI protocol for testing drug efficacy (Prado et al., 2008; Yurayart et al., 2013; Al-Sweih et al., 2014; Leong et al., 2017).

## Discussion

Our knowledge on *Malassezia* yeasts has increased tremendously during the last two decades. Many questions remain ambiguous however, such as their role in pathology, diagnostic procedures, azole susceptibility profiles and clinical implications. *Malassezia* is the major component of the healthy human skin mycobiome but data on the constitution of the skin mycobiome and the abundance of *Malassezia* yeasts directly after birth and during the first years of life are highly inconclusive. Additional studies are needed as a better understanding of early (skin) mycobiome development may aid in understanding transmission routes, and may subsequently aid in infection containment. For example, in cases where *M*. *pachydermatis* was involved, transmission from pets via healthcare workers to patients was suggested which warrants better hygiene measures for hospital staff (Chang et al., 1998; Morris et al., 2005). Interpreting available literature on this topic should be done with caution as variation between studies may arise from methodological differences for factors such as study group size, identification methods and geography.

Concerning the causative species of bloodstream infection, epidemiological surveys suggest that *M. furfur* is the most common species involved. Although further studies are needed to understand the exact mechanisms of this finding; the colonization of skin of patients, as well as the higher virulence of *Malassezia* strains involved in fungemia, might be the driving factors influencing BSI epidemiology.

The occurrence of these infections seems lower among adult patients, and higher among neonatal patients, yet a slanted view exists as cases are likely underestimated, given the special lipid requirement of these yeast species (Iatta et al., 2018). Interestingly neonatal patients seem more frequently colonized with *M. furfur* strains than adults, but more data is needed to support this trend. Although lipid infusion and/or total parenteral nutrition seems to be one of the major risk factors in causing *Malassezia* fungemia due to the lipolytic properties of *Malassezia* yeasts, cases unrelated to intravenous nutrition were also observed (Table 1; Pedrosa et al., 2018; Chen et al., 2019). Interestingly, these infections are usually confined to severely immunocompromised hosts with CVCs, thus confirming the importance of this portal of entry (Iatta et al., 2018; Pedrosa et al., 2018; Chen et al., 2019). Several potential virulence factors have been described and may be involved in *Malassezia* pathogenesis of BSIs. In particular, the ability to produce enzymes such as lipases and/or phospholipases, production of various indolic compounds, the ability to form biofilms, and allergen enriched nanovesicle production were described for *M. furfur* and *M. sympodialis* which are frequently related to fungemia. The properties of these microorganisms to adhere to the skin and to medical indwelling devices by forming biofilms influence the antifungal profile of cells, which might represent another virulence factor in fungemia of severely immunocompromised hosts (Figueredo et al., 2013; Pedrosa et al., 2019a). Interestingly the structure of *Malassezia* biofilms is strain dependent and those of *M. furfur* and *M. pachydermatis* from skin lesions are composed of a more gelatinous extracellular matrix (Figueredo et al., 2013; Angiolella et al., 2018; Pedrosa et al., 2019a). Since the extracellular matrix is directly linked to the virulence of these yeasts, *M. furfur* and *M.pachydermatis* should be more virulent than *M. sympodialis*, which may explain the lower observed incidence of *M. sympodialis* fungemia (Figueredo et al., 2013; Angiolella et al., 2018; Pedrosa et al., 2019a). In recent years, useful new tools for studying virulence and host-pathogen interactions have been developed. *Galleria mellonella* has proven to be a promising *in vivo* infection model (Torres et al., 2020) and two studies showed the potential of a *Agrobacterium tumefaciens* mediated transformation system for studying the role of specific virulence related genes (Ianiri et al., 2016; Celis et al., 2017b). As the clinical outcome of *Malassezia* fungemia does not differ from that of *Candida* yeasts (Iatta et al., 2014a), clinical guidelines need to be organized for an early diagnosis of these yeasts infections. In particular, fever of unknown origin and very high values of C-reactive protein (CRP) should alert the clinicians to suspect *Malassezia* related systemic infections. Diagnostic procedures to recover *Malassezia* organisms from blood are not routinely available, and the most common system used in many laboratories for the detection of bacterial and fungal pathogens (i.e., BacT/Alert system), is ineffective for diagnosing *M. furfur* fungemia (Iatta et al., 2018). Recently, CVC culturing on lipid-supplemented media, has been proposed as a routine procedure to diagnose *M*. *furfur* fungemia in severely immunocompromised patients (Iatta et al., 2018).

Guidelines for the treatment of *Malassezia* spp. skin disorders of pet animals and humans have been assessed, but those related to systemic infections still need to be addressed. Clinical evidence indicated efficacy of azole drugs for the control of skin infections and of AmB for systemic ones (Table 1). However, common recurrences of skin disorders (Negre et al., 2009; Theelen et al., 2018; Bond et al., 2020) as well as the clinical evidence of treatment failure with TER in patients with *pityriasis versicolor*, with KTZ in dogs with otitis (Kim and Pandya, 1998; Gupta et al., 2014) or with FLZ or POS in preventing *M. furfur* fungemia in humans, suggested the occurrence of drug resistance phenomena in these yeast species.

Additionally, the high level of inter- and intraspecies differences of *Malassezia* antifungal profiles might explain the differences in mycological cure rates when an antifungal agent is used to treat what appears to be clinically the same disease state. Different *Malassezia* species might be involved in the same clinical diseases, and/or different genetic types with different antifungal profiles might colonize the same host (Prohic et al., 2015; Velegraki et al., 2015). However, even if the MIC data of *Malassezia* species vary according to the protocol used for susceptibility testing, there are evidences of a very low susceptibility of these yeasts to FLZ, VOR and echinocandins (reviewed in Theelen et al., 2018; Bond et al., 2020). Although *Malassezia* species show differences in their antifungal susceptibility *in vitr*o, the *in vivo* efficacy of antifungal agents needs to be further evaluated by comparing *in vitro* MICs and clinical outcomes. Correlation of high azole MIC values for *Malassezia* spp. with unsuccessful treatment has been reported in some studies (Velegraki et al., 2005; Iatta et al., 2014a; Rojas et al., 2014). The above results need to be further validated with multicentre studies in order to develop therapeutic guidelines. For the moment, it is important to be aware that the genus *Malassezia* comprises a heterogeneous group of species and genotypes that may cause the same pathology, but may vary in their susceptibility to different antifungal agents. Species identification is of paramount importance not only for epidemiological surveillance and outbreak investigation, but also when therapy failure is registered. Interestingly, AmB is among the preferred therapeutic options for the first-line approach, mainly in patients under FLZ prophylaxis (Iatta et al., 2014b). Liposomal-AmB seems to be the most active drug due to the lipophilic nature of these yeasts even if *in vitro* resistance phenomena were registered for L–AmB. The favorable outcome of patients after therapy with micafungin and L-AmB followed by FLZ, might suggest *in vivo* synergism between these drugs as has been previously reported for *Candida* spp. (Rosato et al., 2012; Iatta et al., 2015). Although the observed frequency of systemic *Malassezia* infections is not very high, this may in part be due to underdiagnosis. Use of FLZ prophylaxis with reduced susceptibility for this drug, and the increase of immunocompromised patients, may lead to a higher number of observed *Malassezia* BSIs in the future. Regardless, emerging infections need to be timely diagnosed to aid clinicians in better patient management. The persistence of *Malassezia* yeasts on incubator surfaces and on the hands of health care workers or parents suggested the need for punctilious hygienic measures (Chang et al., 1998; Iatta et al., 2014a,b). The role of lipid infusion in aiding the spread of different *Malassezia* species infections should be also explored. The lack of sufficient literature showing the prevalence of *Malassezia* fungemia, low specificity and sensitivity of different blood culture systems used for the diagnosis of this fungal sepsis, the lack of standardized methods for *in vitro* antifungal susceptibility testing, as well as the lack of studies investigating drug resistance phenomena in these yeasts, call for further studies drafting guidelines for the diagnosis and correct management of *Malassezia* related diseases. Until specific guidelines for diagnosis and treatment of *Malassezia* bloodstream infections are available, clinicians must be aware of the patient population at risk for these infections and they must communicate to the laboratory the need to include special procedures to recover the organisms. Importantly, commonly used culture media in the clinic do not include lipid supplementation, which is needed for *Malassezia* yeasts to grow due to their lipid dependence. Finally, the very low susceptibility of some of these yeasts to azole drugs (i.e., FLZ and VOR) and echinocandins should be considered when a long term or prophylactic therapy is expected to be used. As stated before, *Malassezia* are the major fungal component of the human skin microbiome but for a long time the vast majority of scientific research focused on the role of bacterial microbiota in health and disease. In recent years, the role of fungi attracted more attention regarding their interplay with the human host, but also with other members of the microbiome. Any alteration in either host or microbiota can result in infections and some recent studies emphasized a role for *Malassezia* spp. in other parts of the body, and linked to other diseases known until then (Kong and Segre, 2020). One study reported a role for *M*. *restricta* in Crohn's disease, observing higher relative abundances of intestinal *Malassezia* compared with healthy controls, evoking inflammatory responses through CARD9 signaling (Limon et al., 2019). Though a few recent studies reported dysbiosis signatures of mycobiota in colorectal cancer (CRC) with enrichment of *Malassezia* in CRC compared with controls (Gao et al., 2017; Coker et al., 2019); a new study, for the first time, showed direct proof for the involvement of *Malassezia* in the pathogenesis of cancer. A much increased fungal community in pancreatic ductal adenocarcinomas (PDAs) was significantly enriched for *Malassezia*. Removal of the mycobiome reduced tumor growth, and only repopulation with *Malassezia* accelerated oncogenesis. PDA progression was dependent on mannose-binding lectin (MBL), which binds to glycans of the fungal cell wall to activate a part of the immune system called the complement cascade (Aykut et al., 2019). The relationship between *Malassezia* and the host is complex and more research is needed to understand the various roles that *Malassezia* may express in/on the human host, and the conditions that trigger them. However, the above mentioned recent advancements have paved the way with new tools and insights that may also benefit a better understanding of *Malassezia* bloodstream infections.

## Author Contributions

CC planned, wrote, and contributed to the critical review of the manuscript. WR and BT performed an initial electronic search and drafted and edited the manuscript. CC, BT, TB, and DO performed data cleaning and reviewed the manuscript. CC and BT approved the manuscript for submission. All authors contributed to the article and approved the submitted version.

## Conflict of Interest

The authors declare that the research was conducted in the absence of any commercial or financial relationships that could be construed as a potential conflict of interest.
